# Supplementary material for: Comprehensive RNA-Seq Gene Co-Expression Analysis Reveals Consistent Molecular Pathways in Hepatocellular Carcinoma across Diverse Risk Factors
Source: Biology (Basel). 2024 Sep 26;13(10):765. doi: 10.3390/biology13100765 (PMC11505157; doi:10.3390/biology13100765)
Supplement: Supplementary file 1 [file biology-13-00765-s001.zip › Supplementary Figures.pdf]

Supplementary Figures

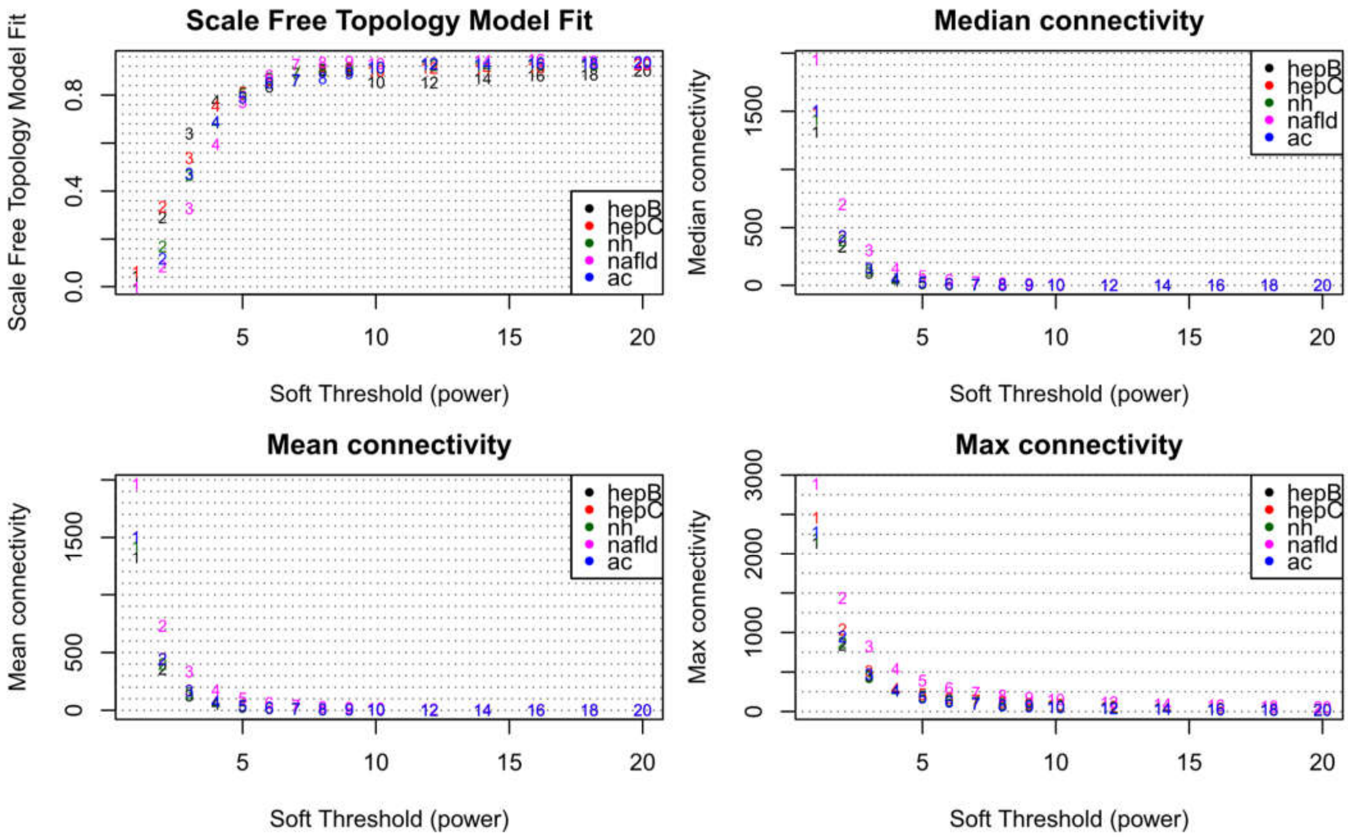

Figure S1. Scale independence and mean connectivity results for the gene expression of each examined etiology of HCC.

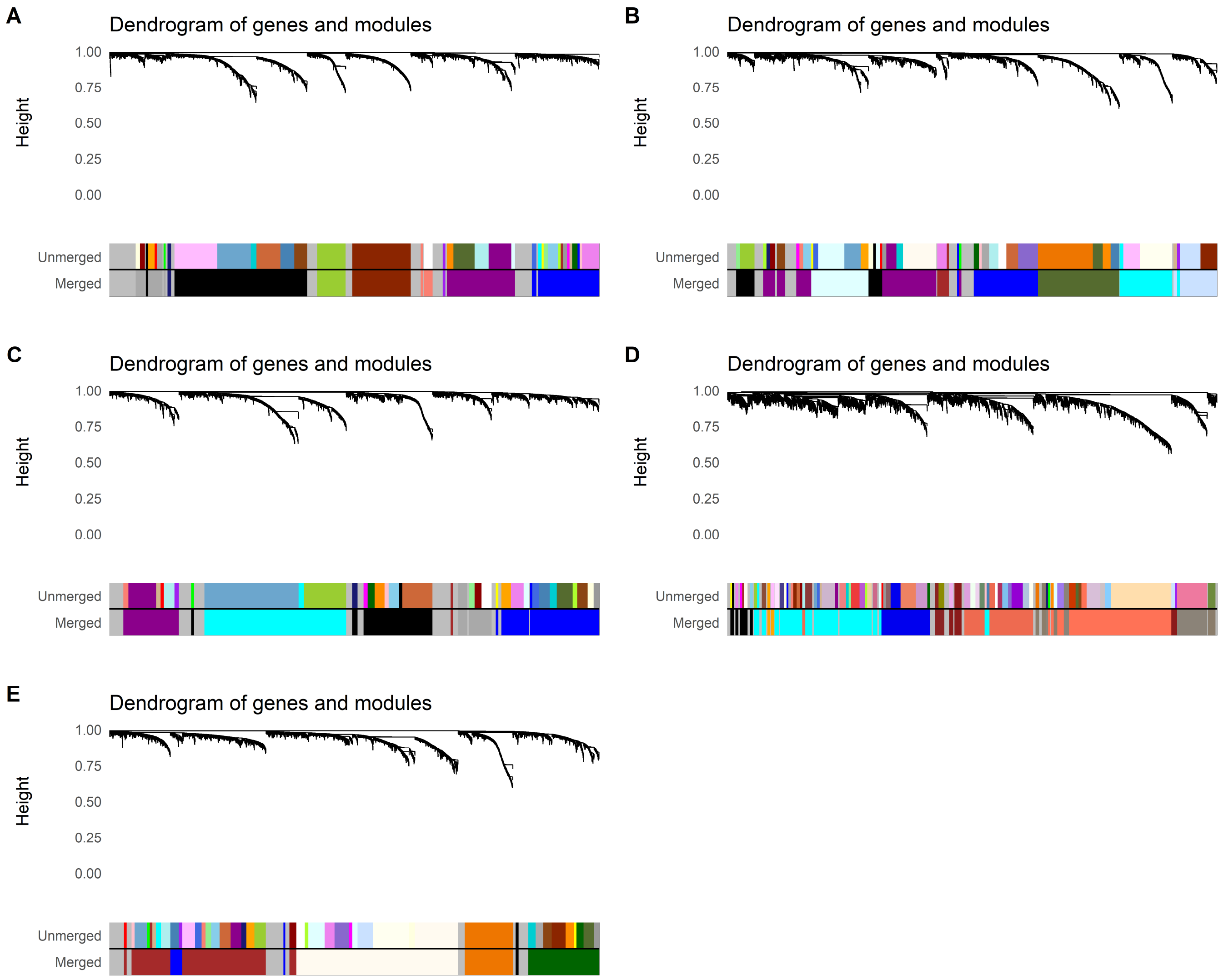

Figure S2. Network Construction Dendrogram Results per Etiology with **(A)** as Alcohol Consumption **(B)** Hepatitis B, **(C)** Hepatitis C, **(D)** NAFLD, **(E)** No History.

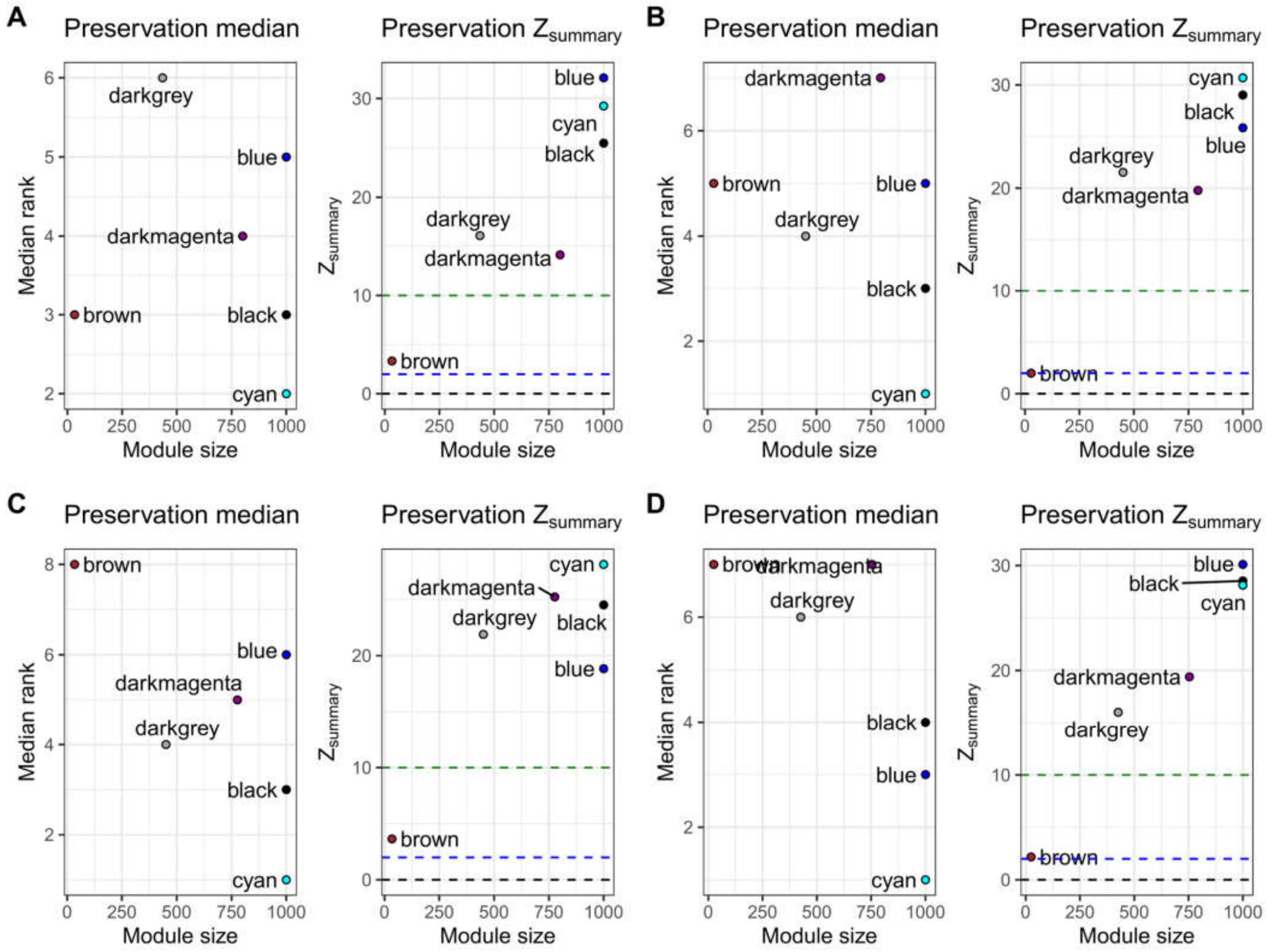

Figure S3. Module preservation results with No History dataset as reference and etiologies **(A)** Alcohol Consumption **(B)** Hepatitis B, **(C)** Hepatitis C, **(D)** NAFLD as the test set.

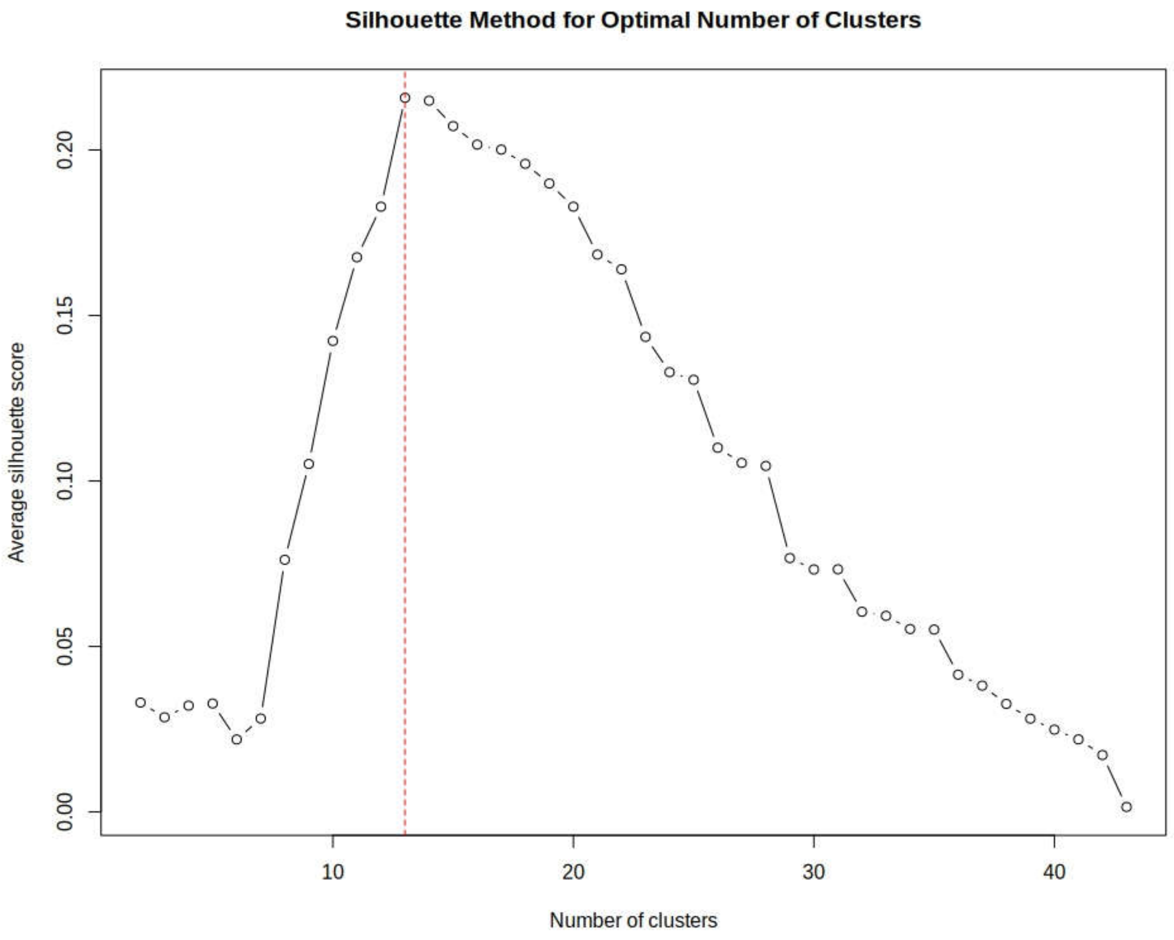

Figure S4. Silhouette method results to determine optimal number of clusters for hierarchical clustering.
